# Two Infectious Agents Causing Hemophagocytic Lymphohistiocytosis

**DOI:** 10.7759/cureus.17947

**Published:** 2021-09-13

**Authors:** Muhammad Umair Atiq, Ahmad Raza, Ammar Ashfaq, Khadija Cheema, Yasir Khan

**Affiliations:** 1 Internal Medicine, Jefferson Abington Hospital, Abington, USA

**Keywords:** hemophagocytic lymphohistiocytosis, secondary hlh, hiv aids, disseminated babesiosis, hematology-oncology

## Abstract

Hemophagocytic lymphohistiocytosis (HLH) is a life-threatening immune activation syndrome that should be recognized earlier for effective treatment. Adults usually have secondary HLH. An uncommon cause of secondary HLH is AIDS and simultaneous opportunistic infections. Acute human immunodeficiency virus (HIV) and opportunistic infections are also independent causes of HLH, so the presence of both should raise suspicion, especially if patients fulfill the criteria. HLH secondary to severe babesiosis is a rare entity as well. Some patients might not meet the full criteria of HLH on presentation, especially when some specific lab test results are still pending. A delay in diagnosis can happen in those cases. Here, we present two cases. The first case is of a 35-year-old homosexual male who presented with constitutional symptoms of one-week duration. He was diagnosed and started on the treatment of HIV. His fever was not resolving and further investigations led to a diagnosis of disseminated histoplasma infection. The patient fulfilled the criteria of HLH as well. Prompt therapy resulted in the improvement of clinical and laboratory parameters. The second case is of a 72-year-old female presenting with fever. A diagnosis of severe babesiosis and secondary HLH was made. Treatment of babesia resulted in the improvement of clinical and biochemical parameters.

## Introduction

Hemophagocytic lymphohistiocytosis (HLH) is a rare syndrome that results from excessive immune activation. The syndrome can be seen most frequently before 18 months of age and less so in children and adults. Children less than three years of age have the highest incidence. The male-to-female ratio is almost 1:1, but there can be male preponderance in adults [1].

HLH can be both sporadic and familial. HLH can also be classified into primary and secondary (reactive) HLH. Primary HLH is associated with underlying genetic mutations and manifests early in life. Secondary HLH usually results from a secondary condition that triggers the immune response. Secondary HLH traditionally is considered to have a better outcome. This system of classification is not precisely accurate. Genetic mutations can be present in both types of HLH. Similarly, a trigger can incite both primary and secondary HLH. In one study, about 14% of adults with HLH had underlying genetic mutations found in familial HLH [2]. Secondary HLH can result due to underlying neoplasia, autoimmune conditions or infections. Here, we present two cases of HLH resulting from underlying infections. To the best of our knowledge, HLH has been rarely reported with these infections.

## Case presentation

Case 1

A 35-year-old male presented through the emergency room (ER) for the evaluation of worsening abdominal pain. His symptoms started one week before the presentation. A week back, he had a constellation of symptoms including sore throat, subjective fever with rigors and chills, night sweats, headache, dry cough and dysuria. A day before the presentation, the patient started having sharp abdominal pain, mostly located in the upper abdomen without any component of radiation. A review of systems was positive for fever with rigors and chills, malaise, sore throat, nausea without vomiting, dysuria without urethral discharge, myalgias and arthralgias.

The patient denied weight loss, changes in vision, sinus symptoms, diarrhea, phlegm production, any new rash, chest pain, or palpitations. Vitals on presentation were a heart rate of 112/minute, respiratory rate of 18/minute, oral temperature of 100.8 °F, blood pressure of 135/86 mmHg and oxygen saturation of 98% while breathing ambient air.

Examination showed an alert and oriented non-toxic-appearing male who was having rigors during the encounter. Eye and oral examinations were normal. Cardiovascular examination revealed a regular heart rate, and normal first and second heart sound with no murmur. Lungs were clear to auscultation bilaterally. Skin examination showed some hyperpigmented lesions on anterior tibial area bilaterally. The patient attributed those lesions to injury during work. No joint effusions were appreciated. Neurologic exam revealed a normal cranial nerve exam with no focal weakness. Gastrointestinal examination revealed some tenderness in the mid-abdomen. Renal punch did not elicit any tenderness. Hepatosplenomegaly was appreciated. Bowel sounds were normal. Genitourinary examination showed no penile lesions, no discharge and no perirectal lesions. No cervical or axillary lymphadenopathy was appreciated. Admission labs are shown in Table 1.

**Table 1:** Labs on admission K, 10^3; ALT, alanine aminotransferase; AST, aspartate aminotransferase; BUN, blood urea nitrogen

Lab	Results
Lactic acid	1.5 mEq/l
BUN	10 mg/dl
Creatinine	0.79 mg/dl
Sodium	133 mEq/l
Potassium	4.4 mEq/l
Chloride	102 mEq/l
AST	154 U/l
ALT	156 U/l
Alkaline phosphatase	126 IU/l
Total bilirubin	0.8 mg/dl
Serum calcium	9.0 mg/dl
White cell count	2.8 K/ul
Absolute neutrophil count	1.7 K/ul
Absolute lymphocyte count	0.9 K/ul
Hemoglobin	13.6 g/dl
Platelet count	131 K/ul
Hepatitis C screening test	Positive
Hepatitis C polymerase chain reaction test	Negative
Hepatitis B core IgM antibody	Non-reactive
Hepatitis B surface antigen	Non-reactive
Hepatitis A IgM antibody	Non-reactive
Hepatitis A antibody	Non-reactive
Infectious mononucleosis screen	Negative

The patient had no diagnosed medical condition, no known past surgical history and was not on any medications. The patient was a single homosexual male who had a high-risk sexual encounter about three weeks before the current presentation. He reported using condoms and denied performing oral sex. He worked as a painter and travel history revealed that he traveled to the United States from Brazil five years ago. There was no history of incarceration, smoking or illicit drug use. His family history was significant for diabetes in the mother and coronary artery disease in his father. The patient had no known allergies.

**Figure 1:**
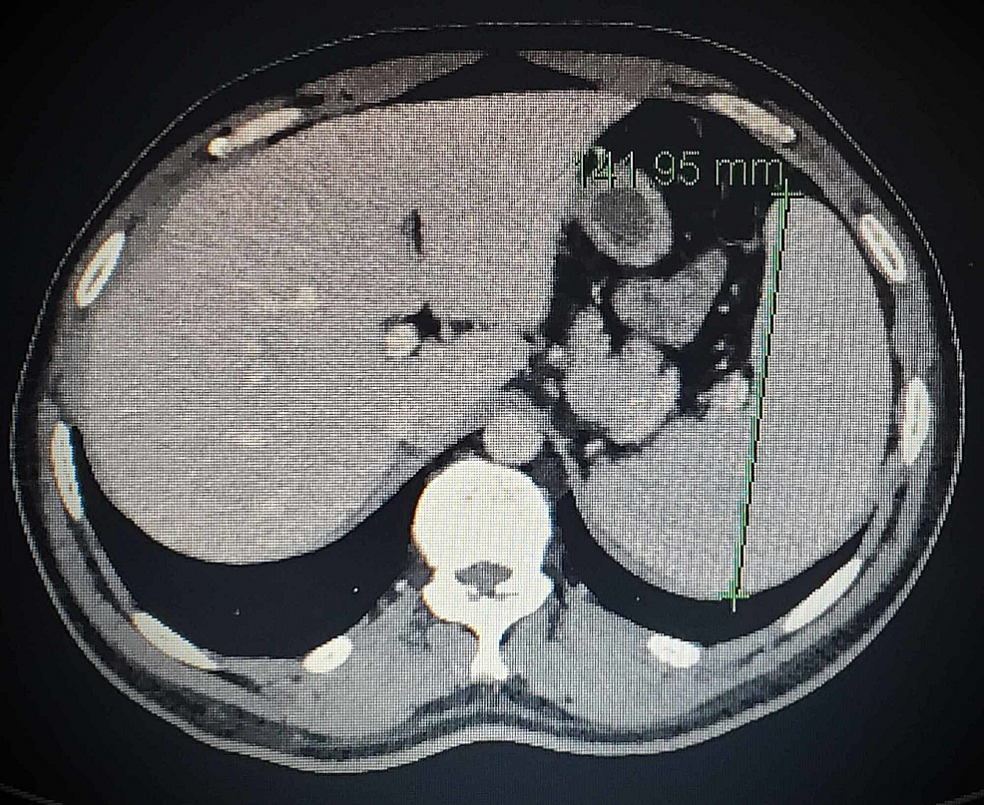
Cross section of the abdomen/pelvis CT scan with contrast showing splenomegaly (spleen measurement shown with a green line)

A CT scan of the abdomen pelvis with contrast showed hepatosplenomegaly (Figure 1). Retroperitoneal adenopathy was found with fat edema, and mild central mesenteric adenopathy. Ultrasound of the abdomen, chest x-ray and CT scan of the thorax with contrast did not show any abnormality.

The patient continued to have a high-grade fever. A human immunodeficiency virus (HIV) detection panel was sent. Tests for Epstein-Barr virus, Cytomegalovirus, syphilis (treponemal and non-treponemal), interferon-gamma release assay, Ehrlichia, Babesia, Anaplasma and serum Histoplasma tests were negative. A transthoracic echocardiogram showed no evidence of valvular vegetations. The patient's labs revealed a positive HIV-1 antibody and raised HIV RNA by polymerase chain reaction (PCR). Table 2 shows HIV-associated labs.

**Table 2:** HIV-related labs HIV, human immunodeficiency virus; PCR, polymerase chain reaction

Test performed	Result (normal range)	Comment
HIV-1 antibody	Positive	
HIV-2 antibody	Negative	
HIV-1 RNA quantitative PCR	249,000 copies/ml	Very high
HIV-1 RNA quantitative PCR	5.40 log copies/ml	Raised
Absolute lymphocytes count	998 cells/ul (850-3900 cells/ul)	
CD19 cell absolute count	48 (110-660 cells/ul)	Low
CD19 %	5 (6%-29%)	Borderline low
CD3 absolute	870 (840-3060 cells/ul)	Within normal range
CD3 %	87 (57%-85%)	Borderline high
CD4 cell absolute count	36 (490-1740 cells/ul)	Very low
CD4 %	4 (30%-61%)	Very low
CD8 cell absolute count	839 (180-1170 cells/ul)	Within normal range
CD8 %	83 (12%-42%)	High
CD4/CD8 ratio	0.04 (0.86-5.00)	Very low
HIV subtype C		
HIV-1 integrase genotype	Raltegravir, elvitegravir and dolutegravir resistance not predicted	

**Figure 2:**
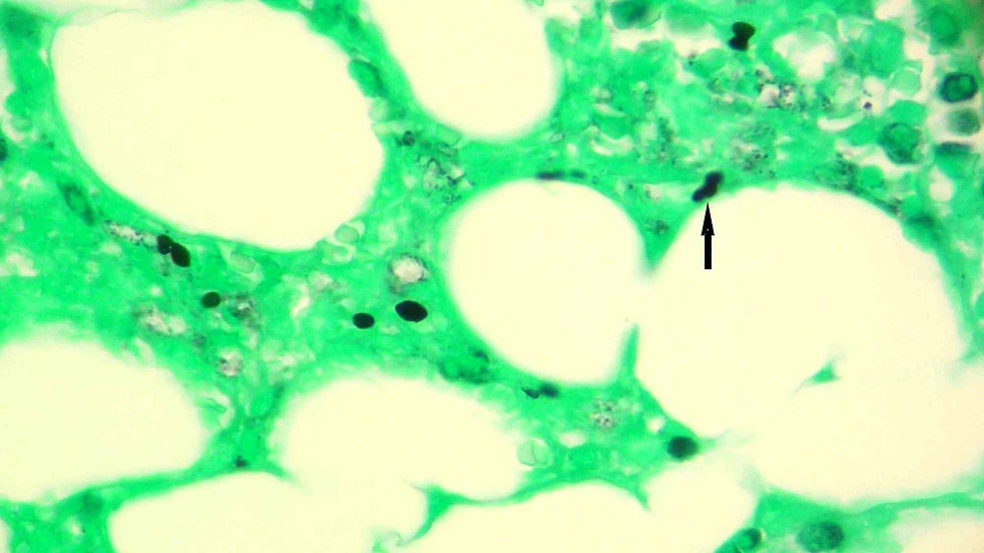
Silver staining of the bone marrow biopsy The picture was taken under oil at 1000x. Histoplasma is shown by the black arrow.

**Figure 3:**
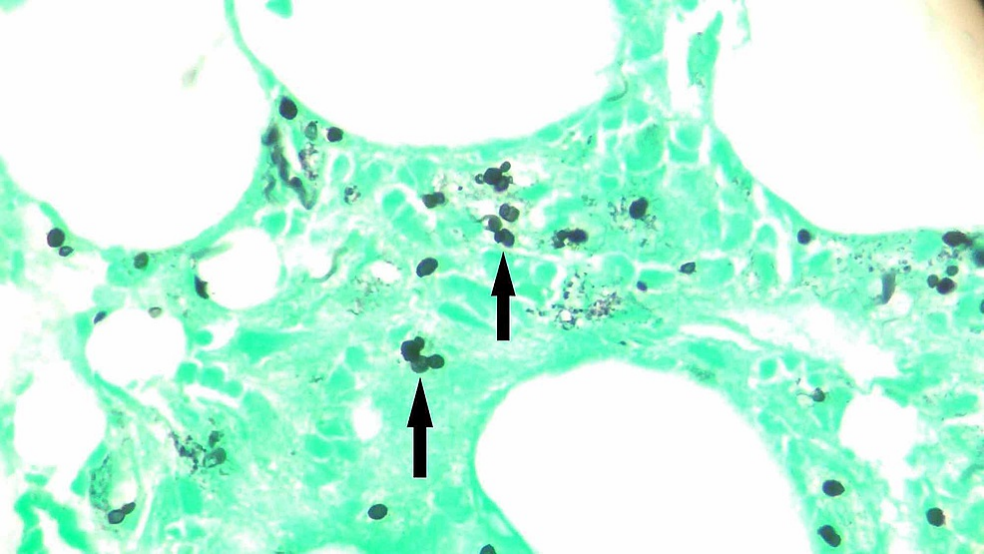
Silver staining of the left para-aortic lymph node biopsy The picture was taken under oil at 1000x. Histoplasma is shown by black arrows.

Antiretroviral therapy was started for the treatment of HIV. Many patients with acute HIV also have opportunistic infections on presentation, which result when testing for Histoplasma and Toxoplasma serum antibodies that are negative [2]. HIV causes secondary HLH and the patient had fever, lymphadenopathy and cytopenia on presentation, so a ferritin level was checked. Ferritin levels were >40,000 ng/ml (normal levels 22-275 ng/ml). Fasting lipid profile showed a serum cholesterol level of 171 mg/dl, triglyceride levels of 275 mg/dl, serum high-density lipoprotein (HDL) level of 8 mg/dl, serum very low density lipoprotein (VLDL) 55 mg/dl and serum low-density lipoprotein (LDL) 108 mg/dl. Diagnostic criteria for HLH also include specific bone marrow findings. The patient underwent bone marrow testing and the results are shown in Table 3. He continued to have spiking fever and during hospital stay became hypotensive at one point. Blood cultures, sputum cultures and peripheral smear revealed no infectious etiology. Para-aortic lymph node biopsy was done. Gram staining of the lymph node showed no neutrophils or organisms. Silver staining showed budding yeast; mucicarmine stain did not show any organism. The silver stain was consistent with a diagnosis of histoplasmosis. Bone marrow and lymph node histology are shown in Figures 2, 3, respectively. The patient was treated with two weeks of amphotericin B as induction therapy followed by itraconazole liquid formulation for maintenance therapy. The patient started showing clinical and biochemical improvement. Repeat ferritin levels were normal and HIV viral load was found to decrease. Cytopenias also resolved and the patient was discharged from the hospital eventually.

**Table 3:** Bone marrow test results AFB, acid-fast bacilli

Bone marrow test	Results
Bone marrow smear, gram staining, AFB smear	No neutrophils, bacteria or AFB detected
Bone marrow aspirate culture	No growth
Pathology of bone marrow	Mild marrow hypocellularity for age with mild myeloid immaturity, a reactive increase in megakaryocytes, focal non-caseating granuloma positive for yeast organisms

Case 2

A 72-year-old female presented to the ER for the evaluation of generalized weakness, rigors, abdominal pain, dark urine and nausea for five to six days. She reported right upper quadrant abdominal pain. The patient saw a tick crawling on her leg a few weeks ago. She denied dysuria, frequency of urine, cough, hemoptysis, recent travel or sick contacts. An evaluation by her primary care physician showed splenomegaly and elevated liver enzymes. Her past medical history was pertinent for coronary artery disease, atrial fibrillation, anxiety, hyperlipidemia, hypothyroidism, mitral valve regurgitation and irritable bowel syndrome, and it included mitral valve repair, coronary artery bypass graft and hysterectomy. She denied tobacco, alcohol or illicit drug use. Her family history was positive for colon cancer in the father, diabetes and heart disease in her mother and breast cancer in her sister. On presentation, her blood pressure was 149/69 mmHg, pulse was 84/minute, respiratory rate was 18/minute, oxygen saturation was 88% while breathing ambient air and she was febrile to 102.4 °F. Physical examination revealed a normal heart and lung and neurological exam was normal. An abdominal exam showed a non-tender, non-distended abdomen. Spleen was palpable in the left upper quadrant.

Admission and subsequent labs are shown in Table 4.

**Table 4:** Lab values of the patient on the first, second, third and last day of hospital stay K, 10^3; ALT, alanine aminotransferase; AST, aspartate aminotransferase; BUN, blood urea nitrogen; LDH, lactate dehydrogenase

Lab test	Admission day results	Admission day 2 results	Admission day 3 results	Labs on discharge
BUN	33 mg/dl	25 mg/dl	27 mg/dl	17 mg/dl
Creatinine	1.03 mg/dl	0.89 mg/dl	0.88 mg/dl	0.85 mg/dl
Sodium	129 mEq/l	133 mEq/l	135 mEq/l	141 mEq/l
Potassium	3.7 mEq/l	4.0 mEq/l	3.6 mEq/l	5.0 mEq/l
AST	238 U/l	372 U/l	458 U/l	51 U/l
ALT	126 U/l	175 U/l	170 U/l	34 U/l
Bilirubin	2.0 mg/dl	2.1 mg/dl	2.8 mg/dl	1.3 mg/dl
White cell count	6.8 K/dl	6.9 K/dl	8.7 K/dl	4.1 K/dl
Hemoglobin	10.2 g/dl (last known normal 14.2 g/dl)	8.6 g/dl	9.0 g/dl	9.6 g/dl (required one pack red blood cell transfusion during her stay)
Platelets	25 K/ul	25 K/ul	35 K/ul	164 K/ul
Absolute neutrophils	5.3 K/ul	5.9 K/ul	9.9 K/ul	Not checked
Absolute lymphocytes	0.9 K/ul	0.5 K/ul	1.4 K/ul	Not checked
Absolute monocytes	0.4 K/ul	0.5 K/ul	0.3 K/ul	Not checked
LDH	Not checked	2490 U/l	3168 U/l	280 U/l
Haptoglobin	Not checked	3	4	<2
Retic count	Not checked	6.2%	14%	3%
Ferritin	Not checked	>20,000 ng/ml	>40,000 ng/ml	1365 ng/ml
Triglyceride levels	Not checked	359 mg/dl	Not checked	Not checked
Parasitemia	6%	7%	19%	No parasite seen

**Figure 4:**
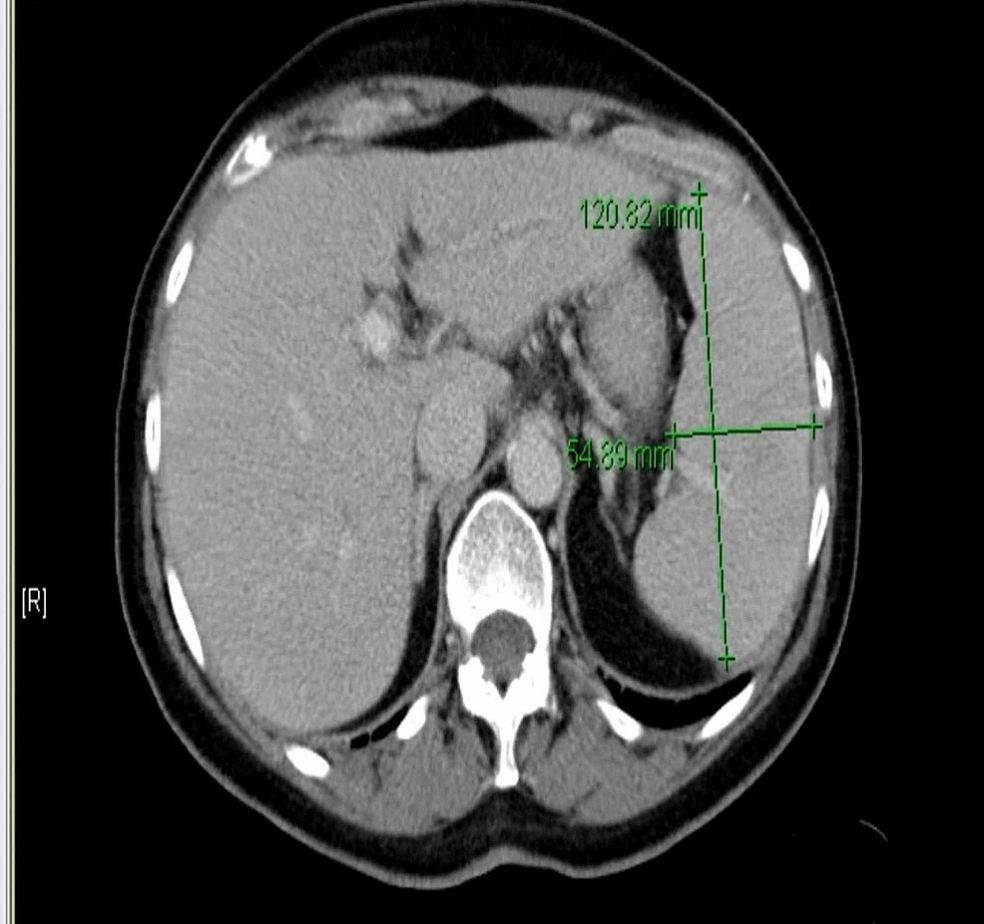
Cross section of the abdomen/pelvis CT scan with contrast showing splenomegaly (spleen measurement shown with green lines)

**Figure 5:**
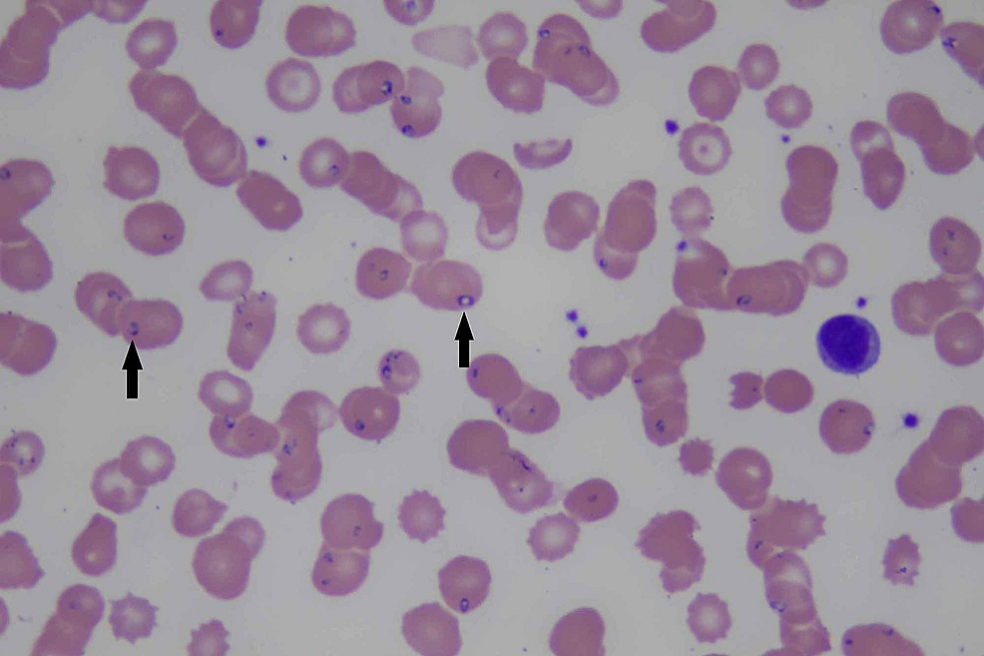
Peripheral smear showing Babesia in red blood cells (shown by black arrows)

Urinalysis showed negative leukocyte esterase, nitrites, ketones and white blood cells. Urobilinogen was positive. A CT scan of the abdomen/pelvis with contrast showed mild splenomegaly but no lymphadenopathy (Figure 4). Due to suspicion of parasitic infection, a peripheral smear was done which showed "maltese cross" appearance within red blood cells. The peripheral smear is shown in Figure 5. The patient was started on intravenous azithromycin and atovaquone to treat babesiosis.

The patient had respiratory distress, hypoxia and continuous fever requiring observation in an intensive care unit. Due to the high degree of parasitemia, she required an exchange transfusion. She met partial criteria for HLH features, so a bone marrow biopsy was done that showed a few histiocytes exhibiting hemophagocytosis. A plan was made to start steroids with or without etoposide in case there was minimal improvement. The patient responded to exchange transfusions and improved over time. Her labs before discharge are also shown in Table 4.

## Discussion

HLH is an immune activation syndrome that can be life threatening. Macrophages, cytotoxic T lymphocytes (CTLs) and natural killer (NK) cells are thought to play a role in its pathology. NK cells and CTLs' cytotoxic function is impaired. Impaired function combined with excessive macrophage activation results in excessive cytokine production, also known as a cytokine storm, which might be the cause of end-organ damage [3-8]. Diagnosis is challenging and often difficult in the face of overlapping symptoms with other diseases. Infections are one of the most common causes of HLH. A variety of infections have been linked to the condition as shown in Table 5. The list is not exhaustive. Many case reports have linked several other organisms to HLH. Conversely, it may be under-diagnosed with certain diseases due to the overlap of symptoms and recovery with the treatment of underlying conditions.

**Table 5:** Infectious causes of secondary hemophagocytic lymphohistiocytosis

Causes	
Viral	Epstein-Barr virus, cytomegalovirus, parvovirus, herpes simplex virus, varicella-zoster virus, measles virus, human herpesvirus 8, H1N1 influenza virus, echovirus, human immunodeficiency virus
Fungal	Histoplasma
Bacterial	Brucella, *Mycobacterium tuberculosis*
Parasital	Babesia, malaria, leishmania

The HLH-2004 trial provides us with diagnostic criteria [9]. The caveat to the diagnostic criteria is its derivation from the pediatric population. Modified diagnostic criteria help to identify patients that do not manifest all the signs, symptoms and laboratory findings of HLH [9,10].

HLH is a rare but increasingly recognized presentation of acute HIV infection with or without disseminated histoplasmosis. There are only a handful of reported cases in the literature. Both HIV and histoplasmosis are independent triggers for HLH and unfortunately our patient had both. Since HLH is a rarely reported life-threatening complication of acute HIV infection, early recognition and prompt treatment is the cornerstone of management. Often, the most significant barrier to treatment and a successful outcome for individuals with HLH is a delay in diagnosis. Several aspects of the clinical presentation of HLH contribute to this delay, including the rarity of the syndrome, the variable clinical presentation, and the lack of specificity of clinical and laboratory findings. Subtle clues in history in the form of risk factors and nonspecific presentation should prompt the clinicians for further evaluation. Our patient had risk factors for HIV, such as a recent high-risk sexual encounter. Once his HIV status was confirmed, further investigation targeted to look for opportunistic infections, especially in the setting of persistent pancytopenia, which led to the diagnosis of disseminated histoplasmosis on bone marrow and lymph node biopsy. What is essential to recognize is the fact that a patient's histoplasma antibody test can be negative in the setting of concomitant immunodeficiency, as in our case, and requires further investigation if there is a high index of suspicion. The clinical findings of fever, cytopenias, hepatosplenomegaly, hypertriglyceridemia and hyperferritinemia fulfilled the criteria for HLH. Bone marrow studies did not show characteristic findings of HLH. One study showed the bone marrow sensitivity to be around 60% in which case clinical judgment should still be used [11]. Our patient was started on HIV treatment consisting of emtricitabine/tenofovir, darunavir and ritonavir along with amphotericin for histoplasmosis. Subsequently, his amphotericin was changed to oral itraconazole. He continued to recover both clinically and biochemically with rapid improvement and eventual recovery of pancytopenia. He was discharged from the hospital with a close follow-up in an HIV clinic. It is also worthwhile to note that disseminated fungal infection can cause some of the abnormalities seen on blood work. While this may make diagnosing HLH challenging, the initial management is to treat the underlying infection.

Human babesiosis is a zoonotic disease that spreads through the bite of an Ixodes tick. The disease can present as mild febrile illness or more severe illness with auto-immune hemolysis and disseminated intravascular coagulation. Severity of illness, host immune status and parasitemia in blood dictates the treatment. Treatment includes antiparasitic drugs as well as addition of plasmapheresis in severe disease. Babesiosis can cause a variety of hematological abnormalities. HLH and splenic rupture are fatal abnormalities and should be recognized timely [12]. HLH due to Babesia infection is rare, and there were less than 10 case reports found by our literature search. Severe babesiosis occurs mostly in immunocompromised patients. There are reports of infection in immunocompetent hosts as well. In all the cases, the treatment of babesia resulted in the resolution of abnormalities attributed to secondary HLH.

The ideal therapy for patients with HLH remains unknown. The HLH-2004 guidelines recommend that primary HLH be treated with an induction phase of steroids, cyclosporine A, and etoposide for eight weeks [9]. Secondary HLH resolves with the treatment of causative agents; however, HLH-specific treatment might be required in select cases.

## Conclusions

Infections are one of the most common causes of secondary HLH. HIV and babesiosis are rarely reported causes of HLH and should be suspected if a patient meets partial HLH-2004 criteria. Full diagnosis might require a bone marrow biopsy. Regardless of the cause, the first step of treatment is the treatment of inciting conditions. Hematology evaluation should be considered in patients not improving despite the treatment of the underlying condition.
